# Premature Ventricular Complex Causing Ice-Pick Headache

**DOI:** 10.1155/2017/3879127

**Published:** 2017-03-07

**Authors:** Selcuk Ozturk, Ertan Yetkin

**Affiliations:** ^1^Ankara Education and Research Hospital, Department of Cardiology, Ankara, Turkey; ^2^Private Yenisehir Hospital, Department of Cardiology, Mersin, Turkey

## Abstract

Ice pick headache is a momentary, transient, repetitive headache disorder and manifests with the stabbing pains and jolts. The exact mechanism causing this disease is unknown. Premature ventricular contractions are early depolarization of the ventricular myocardium and in the absence of a structural heart disease, it is considered to be a benign disease. In this report, we describe a male patient presenting with the symptom of momentary headache attacks accompanied with instant chest pain which is associated with premature ventricular contraction.

## 1. Introduction

Ice-pick headache (IPH), which is also named as “primary stabbing headache” was first defined by Lansche as “ophthalmodynia periodica.” The exact prevalence is unknown but %2–30 of the adult population is thought to be affected in their lifetime [1]. It is characterized with stabbing, momentary headache attacks lasting seconds, being repetitive and transient, and manifestation of multiple jolts. It has a female dominance. The main pathophysiological mechanism underlying this type of headache is unknown [1, 2].

Premature ventricular contraction (PVC), which is also named ventricular extrasystole, premature ventricular beat, or ventricular ectopia, is early depolarization of the ventricular myocardium [3, 4]. Its prevalence varies from %1 to %4 in the general population and increases with aging. Occasionally, it is associated with structural heart disease and increases risk of sudden death. In the absence of a heart disease, it has an excellent outcome [4].

Symptoms in PVCs patients range from palpitation, dyspnea, chest discomfort, lightheadedness, dizziness, exercise limitation, presyncope, syncope, heart failure, and sudden death [4, 5]. Here we describe a case of a PVC patient presenting with the complaint of simultaneous instant chest pain and momentary headache.

## 2. Case

A 78-year-old man presented to cardiology clinic with complaints of instant chest pain accompanied with instantaneous headache at the left temporal region of the head with a strike of lightning. He had been suffering from this complaint for one week. He denied palpitation, dyspnea, syncope, or presyncope. In his detailed history, he had no hypertension, diabetes mellitus, and smoking. He underwent coronary angiography one year ago because of an anginal chest pain and there was no significant stenosis in the coronary arteries. He had been prescribed acetylsalicylic acid 100 mg, atorvastatin 10 mg, and metoprolol 50 mg at that time and the patient was compatible with his treatment. Cardiac examination revealed normal blood pressure, normal heart sounds with a regular rhythm, and no murmurs, S3 or S4. The electrocardiography revealed normal sinus rhythm with a single PVC on the ECG record and during the electrocardiographic examination he experienced the same instant chest pain and headache attack again. During the attack, he had no neurological or gastrointestinal signs. Neurological examination and radiological evaluation including cranial tomography and MR were found to be normal, previously. Echocardiographic examination was within the normal limits and there were no abnormal findings in the blood tests. Then, twenty-four-hour rhythm Holter monitoring was planned and he was told to record chest pain and headache attacks time during the day. Holter monitoring revealed normal sinus rhythm with 80 PVCs most frequently occurring during the night time, synchronous to the patient's chest pain and headache attacks. The patient's average heart rate was 63 beats per minute. Metoprolol therapy was reduced to 25 mg per day and amiodarone therapy has been added to the medical treatment of the patient which were thought to reduce the frequency of the attacks or terminate it. As thought, his complaints significantly improved at the end of the one month and there were no findings of PVC on the ECG recording and rhythm Holter monitoring. He has been followed up and was symptom-free for 6 months. Written informed consent was obtained from the patient.

## 3. Discussion

To the best of our knowledge, this is the first case of a PVC presenting with headache attacks described in literature. IPH is the shortest lasting headache known and it usually occurs without other symptoms. There is not a linkage between IPH and other neurological disorders [1, 2]. Among the various types of headache, migraine is known to be associated with cardiac diseases and cardiac anomalies [6]. In terms of arrhythmia, migraine headache has been reported to be associated with atrial fibrillation in the literature [7, 8]. Recurrent atrial fibrillation attacks have been shown to coincide with migraine headaches [8]. However, there is not any report in the literature about the association between PVC and headache.

Stabbing pains are more dominant in the branches of trigeminal nerve such as orbital, temporal, and parietal regions. Although there is not an exact mechanism in the pathophysiology of IPH, irritation of the trigeminal or other nerves has been suggested as a possible mechanism [1]. This situation can explain the stabbing headache in the temporal region of the head and accompanying strike of lighting in our patient.

Extraordinary symptoms such as burping, tinnitus, and absence of seizure-like attacks have been documented during supraventricular tachycardia [9–11]. The possible underlying mechanism of extraordinary symptoms during supraventricular tachycardia has been supposed to be cross-talk between the cardiac afferent fibers and cranial nerves or ganglia through the impulse propagation [12].

Turhan et al. have described a case of a patient presenting with migrainous headaches and atrial fibrillation attacks. In their patient, they have proposed the possible mechanism causing atrial fibrillation to be the activation of the autonomic nervous system and vagal nerve stimulation during vomiting triggered by migraine attacks [8]. Although this is one of the acceptable explanations, this suggestion cannot be adapted to our patient directly. Cross-talk between the cardiac afferent nerves and cranial nerves through cervical ganglia and spinal cord might have resulted in sensation of instantaneous headache during the PVCs. Several examples of cross-talk as a possible cause of neurological symptoms have been reported and discussed in literature [10–13]. All the fibers forming the different cardiac plexus present shunts with cranial nerves via cervical plexus and brachial plexus [14]. The stimuli originated from the ventricular wall due to PVC and its cross-talk during the impulse propagation might have resulted in a short, momentary headache.

In conclusion, we have described the first case of PVC presenting with momentary headache accompanying instant chest pain which has not been reported in literature, previously. Cardiac arrhythmias should be kept in mind in differential diagnosis of atypical neurological symptoms unless otherwise diagnosed by neurologists.
